# A timely update of global COVID-19 vaccine development

**DOI:** 10.1080/22221751.2020.1838246

**Published:** 2020-10-30

**Authors:** Linda S. Klavinskis, Margaret A. Liu, Shan Lu

**Affiliations:** a Department of Infectious Diseases, King’s College London, London, UK; b ProTherImmune, Lafayette, CA, USA; c Department of Microbiology, Tumor and Cell Biology, Karolinska Institutet, Stockholm, Sweden; d Department of Medicine, University of Massachusetts Medical School, Worcester, MA, USA

**Keywords:** COVID-19, SARS-CoV-2, vaccine, international society for vaccines

## Abstract

This commentary provides an overview and links to presentations of a recent virtual congress series organized by the International Society for Vaccines (ISV) focused on COVID-19 vaccines. The series provided the academic community and vaccine developers as well as the wider general public with balanced information of the global response and resources for COVID-19 vaccines under development featuring: 1) NGOs and the regulatory perspective, 2) the status of vaccine development efforts, and 3) panel discussions to present and discuss challenges. ISV is a non-profit scientific organization whose members work on all areas relevant to vaccines. ISV plans to host additional virtual symposia including regional meetings and incorporating other topics along with COVID-19 vaccines.

There is an unprecedented need to develop, manufacture, test and distribute safe and effective vaccines to fully control the COVID-19 pandemic. In particular, the efforts to limit the spread of the virus have severely challenged opportunities for global scientific collaboration and information exchange because scientists cannot come together in traditional meeting venues to hear the latest developments, share ideas, and establish new partnerships in person.

The International Society for Vaccines (ISV) launched a virtual congress series www.ISVCongress.org as the leading platform for key COVID-19 vaccine developers to share their progress and for the global vaccine community to contribute their collective expertise and wisdom about broader aspects of the global pandemic vaccine response. The aims were threefold: (1) to provide timely information about the processes put in place by regulatory agencies and NGOs for COVID-19 vaccine development, (2) to present primary data from groups developing vaccines, and (3) to provide a forum for discussion by experts about key challenges that confront the COVID-19 vaccine development process.

From June to Aug, 2020, the ISV organized three monthly virtual congresses using a live video broadcasting platform. Each congress commenced with an opening session with two leading experts presenting virology updates of SARS-CoV-2 or the global efforts by CEPI, NIH, the Bill & Melinda Gates Foundation, and the US FDA. Then at each congress four major vaccine developers presented their progress; these developers came from institutions in North America, Europe, China, and Australia. The final session of each congress ended with a panel discussion focusing on a key question facing the development of COVID-19 vaccines. The full list of speakers and panelists along with the titles of their talks and panel discussion topics can be found in Table 1.

**Table 1.** List of congress speakers and titles of presentations.

Date	Name	Institute	Title of talk/panel discussion
**Keynote Presentations**			
June 22	Kwok-Yung Yuen	The University of Hong Kong	Overview of COVID-19; pathogenesis and epidemiology
	Nick Jackson	CEPI	Overview of COVID-19 Vaccine Development
July 21	Larry Corey	Fred Hutchinson Cancer Res. Center (COVID-19 Prevention Network)	COVID Vaccine Planning: The US Government Approach
	Marion Gruber	US FDA	Regulatory Considerations in the Development and Licensure of COVID-19 Vaccines
Aug 25	Myron Cohen	UNC-Chapel Hill	mAbs for COVID-19: Treatment and Prevention
	Lynda Stuart	Bill & Melinda Gates Foundation	COVID-19 Vaccine: How We Win the Race to Billions of Doses
**Vaccine Product Updates**			
June 22	Kate Broderick	Inovio Pharmaceuticals	Advantages of a DNA-based Approach to the Development of a COVID-19 Vaccine
	Barney Graham	VRC/NIAID/NIH	Rapid COVID-19 Vaccine Development Enabled by Prototype Pathogen Preparedness
	Sarah Gilbert	University of Oxford (Partner: AstraZeneca)	Rapid Progress with Development of ChAdOx1 nCoV-19
	Tao Zhu	CanSino Biological	Development of Adenovirus Vector Based COVID-19 Vaccine
July 21	Greg Glenn	Novavax	Progress with the Full Length Recombinant Spike Protein Nanoparticle Vaccine
	George Gao	China CDC	Development of Inactivated COVID-19 Vaccines
	Hanneke Schuitemaker	J&J / Janssen	The Development of an Ad26-based SARS-CoV-2 Vaccine
	Kena Swanson	Pfizer (Partner: BioNTech)	COVID RNA Vaccine Candidate BNT162b1
Aug 25	Keith Chappell	The University of Queensland	Molecular Clamp Stabilized Recombinant Protein Subunit Vaccine for COVID-19
	John Shiver	Sanofi	Recombinant protein and mRNA Vaccine Candidates Against COVID-19
	Jacqueline Miller	Moderna	Moderna’s Coronavirus Vaccine: Early Clinical Data and the COVE Phase III Efficacy and Safety Study
	Brian Ward	Medicago	Development of Plant-Derived SARS-CoV-2 Virus-Like Particle (CoVLP) Vaccine
**Key Issue Panel Discussions**			
June 22			
	Challenges for COVID-19 Vaccines Development: Are Human Challenge Studies Acceptable?		
	Peter Openshaw	Imperial College London	
	Stanley Perlman	University of Iowa	
	Stanley Plotkin	VaxConsult	
July 21			
	Challenges for COVID-19 Vaccines Development: The Roles of Animal Models		
	Bart Haagmans	Erasmus University	
	Vincent Munster	RML/NIH	
	Linda Saif	The Ohio State University	
Aug 25			
	The Role of T and B Cell Responses and Vaccine Assay Standards for Determining Efficacy		
	Alessandro Sette	La Jolla Institute of Immunology	
	Michel Nussenzweig	The Rockefeller University	
	Neil Almond	The National Institute for Biological Standards and Control (NIBSC), UK	

ISV members, as leaders in the global vaccine field, have played key roles in many COVID-19 vaccine programmes. Several speakers and panelists who participated in the congress series are ISV members or ISV Fellows, for example: David Weiner, John Shiver, Stanley Plotkin, and Stanley Perlman from the US, Denise Doolan from Australia, Anna-Lise Williamson from South Africa, Manon Cox from the US/Netherlands, Sarah Gilbert from the UK, and Xavier Saelens from Belgium.

The timely and detailed information presented at the ISV Virtual Congresses highlighted a promising outlook for the field, that multiple candidate vaccines are moving through the clinical trial pipelines. Added to that, well-organized clinical trial systems and clear regulatory review guidelines are in place. That said, any COVID-19 vaccine will need to safeguard the integrity and quality of the vaccine development process, as well as demonstrate safety and efficacy before it can be finally licensed for general public application. The availability of such vaccines to the global population will ultimately determine whether the COVID-19 pandemic can be fully controlled. The detailed programmes of ISV virtual congresses can be found at the ISV Congress website: www.isvcongress.org and the recordings of presentations are available at the YouTube link https://www.youtube.com/channel/UC9_-f8tDAqOmVEZWqGeuFow

The ISV congress series received unprecedented global attention from both vaccine professionals and the general public. The sum total of registered participants was over 6000 and as many as 1800 participants attended each congress in real-time. Global partners, including both public and private organizations, supported or sponsored the organization of the ISV virtual congresses (www.isvcongress.org). Dr. Nick Jackson from CEPI and Dr. Susan Barnett from the Bill & Melinda Gates Foundation (BMGF) kindly served as senior advisors to our congresses.

In summary, the ISV virtual Congress series has further demonstrated the leadership position of the ISV as the only global organization for vaccine professionals. We welcome more vaccine scientists and developers joining ISV (www.isv-online.org) to enable continued and increased scientific collaboration as we collectively strive to prevent suffering and loss of life from preventable diseases. The ISV will continue organizing and supporting future virtual Congresses that have both a broader focus (such as on influenza and COVID-19 vaccines) and a country/regional focus for meetings organized by partner country-based/regional vaccine societies and groups which will facilitate participation due to language and time zone challenges. While ISV membership is truly international, we also support local societies while simultaneously encouraging vaccine scientists to join ISV for increasing global interactions.

